# The Impact of Low-FODMAPs, Gluten-Free, and Ketogenic Diets on Gut Microbiota Modulation in Pathological Conditions

**DOI:** 10.3390/nu11020373

**Published:** 2019-02-12

**Authors:** Sofia Reddel, Lorenza Putignani, Federica Del Chierico

**Affiliations:** 1 Human Microbiome Unit, Bambino Gesù Children’s Hospital, IRCCS, 00165 Rome, Italy; sofia.reddel@opbg.net (S.R.); lorenza.putignani@opbg.net (L.P.); 2 Parasitology Unit, Bambino Gesù Children’s Hospital, IRCCS, 00165 Rome, Italy

**Keywords:** microbiota, dietary patterns, low-FODMAPs diet, ketogenic diet, gluten-free diet

## Abstract

The gut microbiota performs several essential protective, structural, and metabolic functions for host health. The maintenance of a beneficial microbiota requires a homeostatic equilibrium within microbial communities, and between the microorganisms and the host. The gut microbiota composition may be affected by external factors, among them diet habits may be considered most important. In some pathological conditions such as irritable bowel syndrome (IBS), celiac disease (CD), or neurological disorders (ND), specific dietary regimens as low-fermentable, oligo-, di-, mono-saccharides and polyols (FODMAPs), ketogenic (KD), and gluten-free (GFD) diets are considered therapeutic. These kinds of diets are characterized by a reduction or exclusion of a specific nutrient from the entire dietary pattern. Despite these alimentary regimens showing beneficial effects on disease symptoms, they can affect microbiota composition, especially if they are protracted for a long time. To date, only a few studies have reported the effects of these diets on gut microbiota. In this review, we discuss the effects of low-FODMAPs, KD, and GFD on gut microbiota modulation in pathological conditions, advancing the possibility of depicting a balanced diet and developing personalized dietary intervention protocols.

## 1. Introduction

Microbiota science has flourished in recent decades, and the exciting findings have led to reconsidering the role of the huge number of microorganisms that inhabit the human body and in particular the gastrointestinal (GI) tract. In fact, the gut microbiota performs several essential protective, structural, and metabolic functions for host health. A main function of the microbiota is to inhibit pathogen colonization via several mechanisms, which include direct competition for nutrients, production of antimicrobial molecules and modulation of host immune responses [1]. Gut microbiota has the metabolic capacity to produce and regulate multiple compounds that reach the blood circulation and influence the function of distal organs and systems [2], with specific members that can respond to hormones secreted by the host [3]. Then, for its ability to influence organs and, in turn, to be responsive to the secretions of other host organs, microbiota gains the definition of an organ [4]. Particularly, the microbiota provides host with unique and specific enzymes and biochemical pathways [5]. A large proportion of these metabolic processes are beneficial to the host and are involved in either nutrient acquisition or xenobiotic processing, including the metabolism of undigested carbohydrates and vitamin biosynthesis [5].

The maintenance of a beneficial microbiota requires a homeostatic equilibrium within microbial communities, and between the microorganisms and the host. Failure to achieve or maintain this complex homeostasis, called eubiosis, has led to negative consequences on health, causing intestinal diseases and/or disorders, by a dysbiosis framework of gut microbiota.

The gut microbiota composition may be affected by several factors, among them dietary habits may be considered the most important [6]. Indeed, several studies have investigated nutrients and dietary pattern effects on the gut microbiome under physiological conditions [7].

Nevertheless, distinct dietary regimens in specific pathological conditions, such as irritable bowel syndrome (IBS), celiac disease (CD), or neurological disorders (ND) are considered necessary or adjuvant treatments for patients [8,9,10]. Particularly, low-fermentable, oligo-, di-, mono-saccharides and polyols (FODMAPs) diet, gluten-free diet (GFD) and ketogenic diet (KD) are recommended for IBS, CD, and epilepsy, respectively. These diets are characterized by a reduction or exclusion of a specific nutrient from the entire dietary pattern. In particular, in low-FODMAPs diet, poorly absorbed or indigestible short-chain carbohydrates are reduced; in KD, carbohydrates are reduced; and in GFD, gluten is excluded. Despite these alimentary regimens showing beneficial effects on disease symptoms, they can affect microbiota composition, especially if they are protracted. In fact, the availability of distinct food components favors the selective enrichment of microorganisms capable of exploiting these nutrients and supports microbial metabolic cross-feeding, leading to the maintenance of a microbiota eubiosis [11,12].

The identification of specific eubiotic or dysbiotic profiles under a diet therapy during a specific disease are necessary to develop personalized dietary intervention protocols for patients.

In this review, we highlight the effects of low-FODMAPs diet, KD and GFD, on gut microbiota, in the context of IBS, CD, or ND, also discussing the possibility of restoring eubiotic conditions by probiotic supplementation.

## 2. IBS and Low-FODMAPs Dietary Patterns

IBS is one of the most common functional bowel disorders, characterized by abdominal pain and bloating [13,14]. The etiology is unknown, but the pathogenesis seems to be related to all systems included in bowel function regulation: central nervous, enteric nervous, enteroendocrine, gut immune systems, and the gut microbiota [15]. The study of the interplay between diet, host metabolism function, and gut microbiota could help to further understand the complex pathogenesis of this disorder [16,17].

Foods considered as possible etiopathogenetic and/or exacerbating factors of GI disorders are receiving growing attention in the recent scientific literature, with special focus on molecules present in various food classes that would seem to worsen IBS symptoms. These substances, indicated by the term FODMAP, include short-chain fermentable carbohydrates such as lactose, fructose, fructans, galactans, and polyalcohols (sorbitol, mannitol, maltitol, xylitol, and isomalt). These small and osmotically active carbohydrates are poorly absorbed in the small intestine for a slow transport mechanism or an ineffective/reduced enzymatic activity and are rapidly fermented by the colonic microbiota [18]. One of the mechanisms by which FODMAPs exacerbate GI symptoms in IBS is the increasing of water in the small intestine that leads to abdominal pain and bloating [19].

Moreover, FODMAPs bacterial fermentation produces colonic gases, including hydrogen and methane, resulting in luminal distension, and then in triggering IBS symptoms, specifically in those with visceral hypersensitivity [20,21]. Furthermore, FODMAPs are implicated in intestinal motility and in alteration of colonic volume [22].

Many clinical studies have examined the effectiveness of a dietary restriction of FODMAPs on IBS symptoms. To date, clinical trials suggest that patients with IBS report symptomatic benefit from a low-FODMAP diet [23,24,25].

Based on this, a low-FODMAPs diet is now a widely used dietary pattern in managing IBS [18]. The dietary advice consists of the reduction of FODMAPs daily intake from 15–30 g/day (that is generally detected in the usual diet of IBS patients), to 5–18 g/day during the FODMAPs diet [25]. The diet consists of a 4–8-week restriction, followed by a graded FODMAPs reintroduction to determine tolerance [25].

## 3. Low-FODMAPs Dietary Pattern and Its Impact on Gut Microbiota in IBS

Despite the demonstrated benefic effects, low-FODMAPs diet has generated concerns as it can lead to an undesired modulation of gut microbiota, which has been implicated in the pathogenesis of IBS [18]. Particularly, IBS patients has been shown to have a dysbiotic microbiota [26] and further alteration of microbiota through FODMAPs restriction diet might predispose the patient to additional pathological dysbiosis.

In a randomized controlled trial, microbiota of IBS patients submitted to 4-week dietary intervention was compared with that of an IBS patient with a habitual diet. The authors demonstrated a reduction in concentration and proportion of luminal Bifidobacteria after the carbohydrate restriction [27]. Levels of total bacteria and the amount of *Bacteroides*, *Prevotella*, *Eubacterium rectale*, *Clostridium coccoides*, *Faecalibacterium prausnitzii*, *Lactobacillus* and *Enterococcus* remain unchanged after the intervention [27]. Reduction of Bifidobacteria levels as a consequence of a low-FODMAPs diet were also described in recent randomized controlled trials [24,28,29]. Particularly, in the study of Halmos and colleagues, the intake of FODMAPs was very low if compared with the other trials, resulting in a broader gut microbiota alteration. In fact, besides a decrease of Bifidobacteria, a 47% reduction of total bacterial load and of *F. prausnitzii* and *Clostridium* Cluster IV levels were also observed [28].

In a McIntosh study [29], IBS patients were randomized to a low- (LFD) or high-FODMAP diet (HFD) for 3 weeks. Despite a decrease in Bifidobacteria amounts, an increase in Actinobacteria richness and diversity was registered in the LFD group, compared to the HFD group. The latter was also characterized by a decrease of Firmicutes and Clostridiales levels and by a reduction in microbiota overall diversity [29]. On the contrary, a small uncontrolled study revealed no changes in bacterial species richness and in taxa distribution in gut microbiota of IBS children following a low-FODMAPs diet for a week [30].

Interestingly, two studies have investigated the role of the microbiota as a predictor of symptomatic response to the low-FODMAPs diet [17,31]. In a crossover feeding study focused on IBS children, responders patients were enriched in *Bacteroides*, Ruminococcaceae, *F. prausnitzii* and in metabolic pathways related to carbohydrate metabolism [31].

These results suggest that patients with a microbiota characterized by a saccharolytic metabolic capacity may receive a major benefit from a low-FODMAPs diet. Moreover, in a very recent paper, Valeur et al. suggested that pre-intervention levels of specific gut microbiota biomarkers as *B. fragilis*, *Acinetobacter*, *Ruminiclostridium*, *Streptococcus*, and *Eubacterium* may be associated with higher favorable response to a low-FODMAPs diet. These biomarkers were incorporated into a score scheme and subsequently transformed in a response index that could be a useful tool in disease management [17].

Interestingly, also patients with Non-Celiac Gluten Sensitivity (NCGS) seem to benefit from a low-FODMAPs diet with an improvement of gastrointestinal symptoms [32]. However, in these patients a reduction of beneficial Bifidobacteriaceae and an increase of Lachnospiraceae were observed in their gut microbiota [32] (Table 1).

Evidence from the literature, therefore, suggests that the low-FODMAPs diet provokes marked shifts on taxonomic gut microbiota composition. However, larger studies are needed to further understand if these alterations are detrimental for health and if these effects persist for a long time.

Supplementation of the diet with probiotics could help in maintaining the beneficial component of gut microbiota, especially considering the inverse correlation between Bifidobacteria and the symptomatology of IBS [33]; however, to date there is not sufficient knowledge for choosing the optimal probiotic strain, dose, and treatment timing.

## 4. GFD and CD

CD is a frequent chronic inflammatory enteropathy whose clinical framework is various. Symptoms can be intestinal or extra-intestinal and range from acute or chronic diarrhea, abdominal pain, anemia, weight loss up to changes in mood and behavior, and skin disorders [34].

Genetic predisposition (HLA-DQ2 and DQ8 haplotypes) and exposure to gluten prolamines (i.e., gliadin) are involved in the intricate pathogenesis of CD [35]. Gluten triggers the activation of both innate and adaptive immunity mechanisms [36,37], causing the production of different cytokines and chemokines, responsible for the remodeling and destruction of the intestinal mucosa, finally resulting in villi atrophy [38].

In particular, gliadin peptides are deamidated in the intestinal *lamina propria* by the tissue transglutaminase and bind the HLA class II DQ2/8 molecules of the antigen-presenting cells [35]. The gliadin peptides are responsible for the activation of the T cells, macrophages and dendritic cells, with a consequent secretion of inflammatory cytokines [39,40,41]. It follows the activation of the adaptive immune response through the production of anti-endomysium, antigliadin, and anti-transglutaminase antibodies by B cells that increase intestinal permeability [36].

For patients with CD, a GFD is the only available therapy [42], which implies the exclusion of dietary wheat, rye, barley, and hybrids such as kamut and triticale, all of which contain gluten. In most patients under GFD there is an improvement in clinical manifestations, tissue lesions, and blood values and a lowering of the risk of developing clinical complications associated with CD [10].

## 5. Impact of the GFD on Gut Microbiota Modulation

It is known that gut microbiota is impaired in CD with a reduction in beneficial species and an increase in potential pathogens [43]. Altered gut microbiota and metabolome could play a secondary role in aggravating CD pathogenesis or other diseases in celiac patients by modifying the host immunity and physiology [44].

Several studies have investigated the effect of a GFD on gut microbiota of CD patients (Table 2). 

Preliminary studies reported that in CD patients, after 2 years of GFD, the imbalance of duodenal mucosal microbiota were not completely restored with a worsening in the reduction of bacterial richness [45,46]. In fact, although the relative abundances of some potentially pathogenic bacteria such as *Escherichia coli* and *Staphylococcus* decreased after diet, the beneficial species as *Bifidobacterium* and *Lactobacillus* levels remained low.

Nistal and colleagues [47] found a decrease in *Streptococcus* and *Prevotella* levels in CD patients following a GFD. Moreover, decrease of healthy bacteria such as *Lactobacillus*, *Enterococcus*, and Bifidobacteria and increase of detrimental species such as *Bacteroides*, *Staphylococcus*, *Salmonella*, *Shigella*, and *Klebsiella* are reported by Di Cagno et al., 2011 [48]. Also in a recent study, a low abundance of *Bifidobacterium* species was observed in CD patients following a GFD conducted for at least 2 years [49]. Additional support to the indication that a GFD produces a decrease in beneficial bacterial genera including *Bifidobacterium* and *Lactobacillus* and an increase of Enterobacteriaceae, responsible for gut inflammation, came from a study of De Palma et al., which explored the effect of a GFD on healthy subjects [52]. The main inference of this study was that a GFD modulate gut microbiota composition regardless of the disease and that this modulation is probably due to the reduction of the polysaccharide intake. The GFD diet is, in fact, characterized by a substantial reduction of fructans, which carry out a prebiotic action [53]. A similar conclusion was drawn in another recent study, in which the effect of GFD on gut microbiota modulation was investigated through a short-term period [54].

Moreover, in another study, Di Cagno and colleagues described that a lower ratio of *Bifidobacterium* to *Bacteroides* and Enterobacteria (including *E. coli*), characteristic of CD patients before diet, still persist under a GFD, confirming the hypothesis that a GFD only partially restores the imbalances of gut microbiota [50]. The same conclusions were also reported by Schippa et al. in a study conducted on children before and after a GFD, in which mucosa-associated microbiota was analyzed [51]. Recently, the importance of individual microbiota predisposition to GFD was highlighted. In this study, a cohort of CD patients with a persistent symptomatology was compared with a cohort of patients without persistent symptoms, both treated with a 3-year GFD [55]. The results indicated that a previous dysbiosis in patients produced a persistency of symptoms, even while adhering to a strict GFD. Particularly, a reduction in gut microbiota diversity, higher levels of Proteobacteria and lower levels of Bacteroidetes and Firmicutes characterized the gut microbiota of patients with persistent symptoms [55].

The most consistent observation across these studies is the inability of GFD to restore gut microbiota dysbiosis of CD patient in terms of *Lactobacillus* and *Bifidobacterium* abundances and in some cases to worsen the overall microbiota diversity reduction. The reason is yet not clear; however a hypothesis is that since gluten exerts a prebiotic action, its exclusion in GFD provokes changes in gut microbiota composition also in the absence of disease [53]. For this reason, supplementation of GFD with pre- or probiotics is currently an area of growing interest to improve the clinical management of CD patients under GFD.

## 6. Ketogenic Diet (KD) in Neurological Disorders

The low-carbohydrate, high-fat KD is an effective treatment for epileptic patients that fails in responding to anticonvulsant medications [56]. The efficacy of KD rates, measured in seizure reduction, reaches 50% in adult intractable epilepsy [57]. Moreover, recently KD had an increase of application in other ND including autism spectrum disorder (ASD), Alzheimer’s disease [9], Glucose Transporter 1 Deficiency Syndrome (GLUT1-DS) [58] and multiple sclerosis (MS) [59] and even metabolic syndrome and cancer [9]. The classic KD is based on a ratio of fat to carbohydrate plus protein grams of 3:1 or 4:1, which means that 90% of the energy comes from fat and only 10% from carbohydrate and protein combined mixture [60]. It induces ketone body production through fat metabolism, with the goal of mimicking a fasting state in the body’s tissues, shifting the predominant caloric source from carbohydrate to fat [61].

The mechanism of the KD action in epilepsy remains unclear. However, it has been proposed to be related to an involvement in mitochondrial function alterations, effects of ketone bodies on neuronal function and neurotransmitter release, and an antiepileptic effects of fatty acids, and/or glucose stabilization [62].

## 7. Impact of KD in Gut Microbiota Modulation

Different studies investigated the characteristics and composition of intestinal microbiota during a KD (Table 3).

Ma and co-authors explored the effect of KD on gut microbiome composition and its beneficial effect on neurovascular functions, reducing the risk of neurodegeneration and increased beneficial gut microbiota in young healthy mice [63]. However, the authors described a decreased microbial diversity induced by KD. This is the result of reduced carbohydrate intake, which led to a decrease in polysaccharide content and then to a decrease in many gut microbiota bacteria that produce energy from polysaccharides. Moreover, KD appeared to reduce blood glucose levels and body weight, and to increase blood ketone levels. These effects were correlated with the increase of beneficial bacteria such as *Akkermansia muciniphila* and *Lactobacillus*, SCFA producers, and to the reduction of pro-inflammatory taxa such as *Desulfovibrio* and *Turicibacter* [63].

The KD altered the composition of the gut microbiota also in mouse model of ASD [64]. In this study, a significant decrease in total bacterial abundance in cecal and fecal matter was reported. However, differently from the previous study, these authors reported a reduction in the *A. muciniphila* content in mouse microbiota [64].

Olson et al., using two mouse models for refractory epilepsy, revealed the role of gut microbiota in mediating and conferring seizure protection [65]. In fact, the authors demonstrated that the microbiota depletion via high-dose antibiotic treatment raised seizure susceptibility and incidence, in response to the KD. Moreover, the effect of antibiotic treatment resulted annulled by re-colonization with gut bacteria. KD reduced gut microbiota diversity, while increasing the relative abundance of *A. muciniphila* and *Parabacteroides*, hence potentially recognized as with a role in seizure protection. Moreover, the authors found that diet- and microbiota-dependent seizure protection was associated with the increase of gamma-aminobutyric acid (GABA) in bulk and glutamate in the hippocampus [65].

A study in children with refractory epilepsy after a week of KD showed a reduction in richness of gut microbiota, also revealing an increment in Bacteroidetes and decrement in Proteobacteria after KD. At genus level, *Bacteroides*, *Bifidobacterium*, and *Prevotella* resulted increased after KD, while *Cronobacter* diminished. The authors concluded that KD could alleviate seizure frequency in infants with refractory epilepsy and rapidly alter gut microbiota. In fact, the microbiota of epileptic infants differed from that of healthy controls, and after KD therapy changed significantly, with a decrement in pathogens and an increment in beneficial bacteria [66].

In another study, in children affected by refractory epilepsy, after 6 months of treatment with KD, the fecal microbiota profiles showed lower diversity after KD therapy and revealed significantly decreased abundance of Firmicutes and increased levels of Bacteroidetes. In this study, the subjects differently responded to a seizure reduction. In subjects’ non-responders after KD, Clostridiales, Ruminococcaceae, Rikenellaceae, Lachnospiraceae, and *Alistipes* were enriched compared to effective patients. These results suggested that the changes in gut microbiota composition may be associated with different efficacy after KD, and that a specific gut microbiota may serve as an efficiency biomarker and a potential therapeutic target in patients with refractory epilepsy [67].

In the study of Swidsinski and co-workers, the colonic microbiome was investigated in an auto-immune MS cohort of patients during KD treatment [68]. The effects of a KD in the short term produced a reduction of bacterial concentrations and diversity. After 6 months, an inverse effect was registered with a bacterial richness significantly increased and a restoring in bacterial biofermentative functions, markedly impaired in MS patients. Therefore, it appeared that KD normalized concentrations and functions of the colonic microbiome after 6 months of treatment [68].

In GLUT1 DS patients treated with KD for three months a statistically significant increase in *Desulfovibrio* spp., a sulfite-reducing bacteria, was registered [69]. These effects could be mediated by fat-induced-taurine conjugation of hepatic bile acids, which increases the availability of organic sulfur used by sulfite-reducing microorganisms. Since in the short-term KD treatment the microbiota changing was not accompanied by an amelioration of patient clinical symptoms, the authors suggested that dysbiosis may become important if KDs are consumed for longer periods without supplementing with fermentable substrates, especially considering the potential detrimental effect on gut health of sulfate-reducing bacteria. Therefore, in patients with demonstrated dysbiosis, it may be reasonable to consider a supplementation with pre or probiotics to potentially restore the “ecological balance” of intestinal microbiota [69].

## 8. Probiotics Supplementation

Only a few studies have investigated the supplementation of these dietary patterns with probiotics that could be useful for the recovery and maintenance of a eubiotic gut microbiota.

Particularly, promising evidence comes from studies exploring the effect of a GFD supplemented with probiotics in CD patients [70].

In a recent randomized control trial, Olivares et al. [71] assessed whether the intervention in the gut ecosystem with *B. longum* CECT 7347 improved the efficacy of the GFD in children with newly diagnosed CD. Thirty-three children were randomized to receive a capsule containing the probiotic or placebo daily for 3 months together with a GFD. The coupled intervention with GFD and probiotic led to height percentile increase in terms of growth-related parameters and to a decrease in peripheral CD3+ T lymphocytes number. Interestingly, *B. longum* CECT 7347 induced a significant decrease of *Bacteroides fragilis* and Enterobacteriaceae and an increase of harmless to potentially harmful bacteria ratio [71].

In the study of Quagliariello et al. [72] was evaluated the efficacy of 3 months intake of *B. breve* strains B632 and BR03 on 45 CD patients on GFD. The authors demonstrated an increase of Actinobacteria and a restoration of the Firmicutes/Bacteroides ratio, induced by probiotic supplementation, thus re-establishing the eubiosis of CD children under a GFD [72].

The same probiotic strains *B. breve* B632 and BR03 were administered for three months to 40 children with CD on GFD in another clinical trial, showing a microbiota restoration with an increment in the production of acetic acid and total short-chain fatty acids (SCFAs) [73].

On the contrary, in the study of Harnett et al., based on 45 CD patients under GFD, the supplementation with VSL3 did not produce any differences in the gut microbiota composition and in clinical outcome [74].

Francavilla et al. obtained an improvement in the severity of IBS-type symptoms, in 54 randomized CD patients on strict GFD, receiving for 6 weeks a mixture of 5 strains of lactic acid bacteria and Bifidobacteria: *L. casei* LMG 101/37 P-17504, *L. plantarum* CECT 4528, *B. animalis* subsp. *lactis* Bi1 LMG P-17502, *B. breve* Bbr8 LMG P-17501, *B. breve* Bl10 LMG P-17500. Particularly, authors observed an increase of presumptive lactic acid bacteria, *Staphylococcus* and *Bifidobacterium* in patients receiving probiotic treatment [75].

Moreover, in the only study regarding the supplementation of the low-FODMAPs diet with probiotics, authors administered a multistrain probiotic formulation (*Streptococcus thermophilus* DSM 24731, *B. breve* DSM 24732, *B. longum* DSM 24736, *B. infantis* DSM 24737, *Lactobacillus acidophilus* DSM 24735, *L. plantarum* DSM 24730, *L. paracasei* DSM 24733, *L. delbrueckii* subsp. *bulgaricus* DSM 24734) to 27 randomized IBS patients under a 4 weeks low-FODMAPs diet. The authors observed after the probiotic treatment a restoration of *Bifidobacterium*, always reduced in the gut microbiota of patients under a low-FODMAP diet [24].

Regarding the probiotic treatment coupled to KD, to date there are no reports on this topic.

## 9. Conclusions and Future Perspectives

GFD, low-FODMAPs diet, and KD represent real therapies in distinct pathological conditions. However, many studies have highlighted a pronounced compositional change of the gut microbiota due to these dietary patterns. This side effect seems to be more pronounced in patients under GFD and low-FODMAPs diet, while for KD the picture is not entirely clear. Nevertheless, the data on the effects of long-term GFD on microbiota in CD patients available in the literature are bit dated with respect to the other pathologies.

Considering that gut microbiota has been implicated in the pathogenesis of the diseases here described, its further alteration may have potential consequences for long-term health and might predispose the patient to additional pathological symptoms.

This safety concern constitutes the basis for investigating the supplementation of these diets with probiotics that could be useful for the recovery and maintenance of a eubiotic gut microbiota. For CD and KD, the integration of a GFD and a low-FODMAPs diet with probiotics seems to counteract gut microbiota imbalances and in particular restore *Bifidobacterium* levels. Regarding KD, some authors suggest that a prolonged KD-induced dysbiosis could raise concern and then supplementation with pre- or probiotics could be recommended.

In conclusion, even if further studies are needed to confirm and/or expand these findings, the evidence collected so far are encouraging and the use of dietary patterns coupled to probiotics could be useful to avoid side effects on health due to the alteration of the intestinal microbiota, especially in pathological subjects already characterized by intestinal dysbiosis.

## Figures and Tables

**Table 1:** Main findings related to the effect of low-FODMAPs diet (LFD) on gut microbiota in irritable bowel syndrome (IBS) and Non-Celiac Gluten Sensitivity.

*N* of Subjects	Age of Subjects	Population	Time of Administration	Methodology	LFD	Findings	Year	Authors
19 IBS patients on LFD and 22 IBS patient on habitual diet	18–65 years	IBS	4 weeks	FISH	restriction of foods high in fructans (e.g., wheat products, onions), GOS (e.g., legumes), polyols (e.g., pear, sugar-free gums), lactose (e.g., mammalian milk), and excess fructose (e.g., honey)	↓ Bifidobacteria in LFD versus habitual. No differences in levels *of* total bacteria, *Bacteroides Prevotella*, *E. rectale*-*C. coccoides*, *F. prausnitzii*, *Lactobacillus* and *Enterococcus* after LFD	2012	Staudacher [27]
51 IBS patients on LFD and 53 IBS patients on Sham diet	18–65 years	IBS	4 weeks	qPCR and 16S rRNA-Illumina sequencing	restriction of foods high in fructans (e.g., wheat products, onions), GOS (e.g., legumes), polyols (e.g., pear, sugar-free gums), lactose (e.g., mammalian milk), and excess fructose (e.g., honey)	↓ *Bifidobacterium* spp. in LFD versus sham	2017	Staudacher [24]
37 IBS patients: 19 on LFD, 18 on high FODMAPs (HFD)	LFD group, 50.3 median age (years) HFD group, 51.5 median age (years)	IBS	3 weeks	16S rRNA-Illumina sequencing	restriction of foods high in fructans (e.g., wheat products, onions), GOS (e.g., legumes), polyols (e.g., pear, sugar-free gums), lactose (e.g., mammalian milk), and excess fructose (e.g., honey)	↑ Actinobacteria, Firmicutes, Clostridiales; ↑ecological diversity in LFD versus HFD; ↑ Clostridiales XIII Incertae sedis spp. In addition, *Porphyromonas* spp. in LFD versus baseline; ↓ Propionibacteriaceae and Bifidobacteria in LFD versus baseline	2017	McIntosh [29]
30 IBS randomized to LFD and habitual Australian diet and 8 healthy individuals	IBS 41 median age (years) CTRL 31 median age (years)	IBS	3 weeks	qPCR	LFD: 3.05g (mean value) total FODMAPs. Habitual diet: 23.7 (mean value) total FODMAPs	↓Bifidobacteria, *F. prausnitzii*, *Clostridium* cluster IV *A. muciniphila*, total bacteria in LFD versus habitual diet; ↑ Clostridium cluster XIV diversity in LFD versus habitual diet	2014	Halmos [28]
12 IBS patients	10.9 median age (years)	IBS	1 week	16S rRNA 454 pyrosequencing	restriction of foods high in fructans (e.g., wheat products, onions), GOS (e.g., legumes), polyols (e.g., pear, sugar-free gums), lactose (e.g., mammalian milk), and excess fructose (e.g., honey)	No changes in terms of richness of specie and in the taxa composition after LFD	2014	Chumpitazi [30]
33 IBS children randomized to LFD (16) or habitual American diet (17)	7–17 years	IBS	4 days	16S rRNA 454 pyrosequencing	The low-FODMAP diet contained 0.15 g/kg * day (maximum 9 g/day) of FODMAPs. The habitual diet contained 0.7 g/kg * day (maximum 50 g/day) of FODMAPs	↑ *Bacteroides,* Ruminococcaceae, *F. prausnitzii* and Erysipelotrichaceae in responder patients at baseline	2015	Chumpitazi [31]
61 IBS patients (32 responders and 29 non-responders)	Responders 32.5 median age (years) non-responders 39 median age (years)	IBS	4 weeks	GA-map™ Dysbiosis Test	restriction of foods high in fructans (e.g., wheat products, onions), GOS (e.g., legumes), polyols (e.g., pear, sugar-free gums), lactose (e.g., mammalian milk), and excess fructose (e.g., honey)	↑ *B. fragilis*, *Acinetobacter*, *Ruminiclostridium Streptococcus*, *Eubacterium* in responders versus non-responders at baseline; ↓ Clostridia, *Negativicutes*, Bacilli Actinomycetales, *Anaerotruncus,* Clostridiales *Shigella, Escherichia* in non-responders versus responders at baseline	2018	Valeur [17]
19 NCGS patients 10 CTRL	NCGS 33.8 median age (years) healthy controls 32.8 median age (years)	NCGS	2 weeks LFD Followed by 2 weeks GFD	16S rRNA-Illumina sequencing	LFD in healthy individuals: 0.98 g/day lactose, 0.87 g/day maltose, 0.22 g/day sorbitol LFD in NCGS: 1.10 g/day lactose,10.83 fructose, 0.73 g/day, 0.12 g/day sorbitol	GFD: ↑ Bacteroidaceae ↓ Lachnospiraceae; LFD: ↓Bifidobacteriaceae ↑ Lachnospiraceae	2018	Dieterich [32]

List of abbreviations in alphabetical order: CTRL, controls; FISH, fluorescence in situ hybridization; FODMAP, fermentable, oligo-, di-, mono-saccharides and polyols; GFD, gluten-free diet; GOS, Galacto-oligosaccharides; IBS, irritable bowel syndrome; LFD, low-FODMAPs diet; NCGS, Nonceliac gluten sensitivity; qPCR, quantitative polymerase chain reaction. Bacterial increase: ↑; bacterial reduction: ↓.

**Table 2:** Main findings related to the effect of gluten-free diet (GFD) on gut microbiota in celiac disease (CD).

*N* of Subjects	Age of Subjects	Population	Time of Administration	Methodology	Findings	Year	Authors
16 patients and 8 healthy	5 median age (years)	CD	2 years	qPCR	↓ *E. coli* and *Staphylococcus*	2009	Collado [45]
30 patients and 8 healthy	4.9 median age (years)	CD	1–2 years	FISH and flow cytometry	↑ overgrowth of total and Gram-negative bacteria	2007	Nadal [46]
8 patients and 5 healthy children; 10 patients and 5 healthy adults	Children 5.5 median age and adults 26.3 median age (years)	CD	n.d.	16S rRNA-based metagenomics	↓ *Streptococcus* spp. and *Prevotella* spp.	2012	Nistal [47]
19 patients and 15 healthy	6–12 years	CD	2 years	PCR-DGGE	↓ *Lactobacillus*, *Enterococcus* and Bifidobacteria; ↑ *Bacteroides*, *Staphylococcus*, *Salmonella*, *Shigella* and *Klebsiella*	2011	Di Cagno [48]
14 patients and 42 healthy	adults	CD	at least 2 years	Microbiological culture	↓ *Bifidobacterium*	2014	Golfetto [49]
21 patients	6–12 years	CD	2 years	PCR-DGGE analysis and Microbiological culture	↑ *L. brevis*, *L. rossiae* and *L. pentosus*; ↑ *B. longum*, *B. infantis*, *B. lactis*, *B. dentium*, *B. bifidum*	2009	Di Cagno [50]
20 patients and 10 healthy	11.7 median age (years)	CD	9 months	TTGE	↑ *B. vulgatus* and *C. coccoides* group	2010	Schippa [51]

List of abbreviations in alphabetical order: CD, celiac disease; DGGE, denaturing gradient gel electrophoresis analysis; FISH, fluorescent in situ hybridization; GFD, gluten-free diet n.d.: not defined; qPCR, quantitative polymerase chain reaction; TTGE, temporal temperature gradient gel electrophoresis. Bacterial increase: ↑; bacterial reduction: ↓.

**Table 3:** Main findings related to the effect of ketogenic diet (KD) on gut microbiota.

Subjects/Animals	*N* of Subjects	Age of Subjects	Population	Time of Administration	KD	Methodology	Findings	Year	Authors
C57BL/6 male mice	9–10 for treated and untreated groups	12–14 weeks of age	Healthy	16 weeks	75.1% fat (composed of saturated, monounsaturated, and polyunsaturated fatty acids), 8.6% protein, 4.8% fiber, 3.2% carbohydrates, 3% ash, and less than 10% moisture	16S rRNA-based metagenomics	↓ microbiota diversity; ↑ *Adlercreutzia*, *Lactobacillus*, Erysipelotrichaceae-*Clostridium*, *A. muciniphila*; ↓ *Turicibacter*, Clostridiaceae-*Clostridium*, *Dorea*, *Desulfovibrio*	2017	Ma [63]
C57BL/6 and BTBR^T + tf/j^	21 and 25 per group	5 weeks of age	ASD	10–14 days	75% kcal fat	qPCR analysis	↓ bacterial abundance in cecal and fecal matter; ↓ *A. muciniphila*, *Methanobrevibacter*, and *Roseburia* in cecal matter; ↓ *A. muciniphila* and *Lactobacillus*, ↑ Enterobacteriaceae in fecal matter	2016	Newell [64]
Germ Free wild-type Swiss Webster and SPF C3HeB/FeJ KCNA1 KO mice	Variable for each group	3–4-week-old	6-Hz-induced seizure model of refractory epilepsy	3 weeks	6:1 KD	16S rRNA-based metagenomics	↓ alpha diversity; ↑ *A. muciniphila* and *Parabacteroides*	2018	Olson [65]
Human	14 patients and 30 healthy controls	1.95 median age (years)	Refractory epilepsy	1 week	Zeneca products: lipid-to-non-lipid ratio of 4:1, with 60% of the total lipid long-chain triglyceride and 40% medium-chain triglyceride	16S rRNA-based metagenomics	↓ richness of gut bacteria; ↑ Bacteroidetes and ↓ Proteobacteria; ↑ *Bacteroides*, *Bifidobacterium* and *Prevotella*; ↓ *Cronobacter*	2017	Xie [66]
Human	20 pre and post treatment	4.75 median age (years)	Refractory epilepsy	6 months	4:1 ratio KD	16S rRNA-based metagenomics	↓ richness of gut bacteria; ↑ Bacteroidetes; ↓ Firmicutes and Actinobacteria	2018	Zhang [67]
Human	25 patients 14 healthy controls	n.d.	Auto-immune multiple sclerosis (MS)	6 months	An average daily intake of <50 g carbohydrates, >160 g fat, and <100 g protein was recommended	FISH	↓ total concentration and diversity of substantial bacterial groups at week 2; ↑ total concentration at 24 weeks	2017	Swidsinski [68]
Human	6 pre and post treatment	8–34 years	GLUT1 DS	3 months	Starting from 1:1 to gradually proceed to 2:1, 3:1 or 4:1 ratio KD	qPCR analysis	↑ *Desulfovibrio*	2017	Tagliabue [69]

List of abbreviations in alphabetical order: ASD, autism spectrum disorder; FISH, fluorescence in situ hybridization; GLUT1 DS, Glucose Transporter 1 Deficiency Syndrome; Hz, Hertz KD, ketogenic diet; n.d., not defined; qPCR, quantitative polymerase chain reaction. Bacterial increase: ↑; bacterial reduction: ↓.
